# Life in plastic, it’s fantastic! How *Leishmania* exploit genome instability to shape gene expression

**DOI:** 10.3389/fcimb.2023.1102462

**Published:** 2023-01-26

**Authors:** Jennifer A. Black, João Luís Reis-Cunha, Angela. K. Cruz, Luiz. R.O. Tosi

**Affiliations:** ^1^ Ribeirão Preto Medical School, University of São Paulo, Ribeirão Preto, Brazil; ^2^ The Wellcome Centre for Integrative Parasitology, School of Infection, Immunity, and Inflammation, University of Glasgow, Glasgow, United Kingdom; ^3^ Biomedical research institute, University of York, York, United Kingdom

**Keywords:** *Leishmania*, genome plasticity, replication, adaption, aneuploidy, DNA instability

## Abstract

*Leishmania* are kinetoplastid pathogens that cause leishmaniasis, a debilitating and potentially life-threatening infection if untreated. Unusually, *Leishmania* regulate their gene expression largely post-transcriptionally due to the arrangement of their coding genes into polycistronic transcription units that may contain 100s of functionally unrelated genes. Yet, *Leishmania* are capable of rapid and responsive changes in gene expression to challenging environments, often instead correlating with dynamic changes in their genome composition, ranging from chromosome and gene copy number variations to the generation of extrachromosomal DNA and the accumulation of point mutations. Typically, such events indicate genome instability in other eukaryotes, coinciding with genetic abnormalities, but for *Leishmania*, exploiting these products of genome instability can provide selectable substrates to catalyse necessary gene expression changes by modifying gene copy number. Unorthodox DNA replication, DNA repair, replication stress factors and DNA repeats are recognised in *Leishmania* as contributors to this intrinsic instability, but how *Leishmania* regulate genome plasticity to enhance fitness whilst limiting toxic under- or over-expression of co-amplified and co-transcribed genes is unclear. Herein, we focus on fresh, and detailed insights that improve our understanding of genome plasticity in *Leishmania*. Furthermore, we discuss emerging models and factors that potentially circumvent regulatory issues arising from polycistronic transcription. Lastly, we highlight key gaps in our understanding of *Leishmania* genome plasticity and discuss future studies to define, in higher resolution, these complex regulatory interactions.

## 1 Introduction

Pathogenic organisms can rapidly adapt to challenging environments by altering their genome composition. Mutagenesis, genetic exchanges, abnormal chromosome number (aneuploidy and chromosome instability; CIN), DNA insertions and deletions (indels), single nucleotide polymorphisms (SNPs), gene copy number variations (CNVs), and other DNA rearrangements can give rise to genome heterogeneity and selectable fitness enhancing traits (Merlo et al., 2006; Żmieńko et al., 2014; Lee et al., 2016; Bolhaqueiro et al., 2019; Todd et al., 2019; López et al., 2020; Watkins et al., 2020). For a host, a genetically flexible pathogen has important clinical consequences, including the selection and emergence of drug resistance, ultimately limiting treatment options (Yang et al., 2019; Sah et al., 2021; Kukurudz et al., 2022). Nevertheless, a flexible genome requires limits to prevent the accumulation of deleterious mutations and catastrophic genome collapse. By improving our understanding of how genome plasticity is harnessed in pathogens, we may uncover key targetable dependencies in these processes, ultimately improving the clinical management of numerous medically important infections.

Over the last decade, single cell sequencing (SCS) technologies (Imamura et al., 2020; Louradour et al., 2020; Bussotti et al., 2021; Negreira et al., 2022), novel screening strategies (Baker et al., 2021), improved genetic engineering using CRISPR/Cas9 (Zhang and Matlashewski, 2015; Espada et al., 2021; Beneke et al., 2022) and inducible gene deletion (Duncan et al., 2016; Damasceno et al., 2020b; Yagoubat et al., 2020) have seen the Kinetoplastid parasite *Leishmania*, a single-celled eukaryote, emerge as a strong model of adaptive genome plasticity due to its surprising tolerance for extensive genomic alterations (Rogers et al., 2011; Sterkers et al., 2011; Lachaud et al., 2014; Ubeda et al., 2014). Over 20 species of *Leishmania* cause the vector-borne, neglected tropical disease (NTD) leishmaniasis in humans and animals. Leishmaniasis primarily affects poverty-stricken regions in the tropics and sub-tropics of the world (Torres-Guerrero et al., 2017; Burza et al., 2018), with the symptoms and disease outcomes partially determined by the infecting species. Broadly, the disease manifests as one of two main forms: tegumentary and visceral leishmaniasis. Tegumentary leishmaniasis includes Cutaneous Leishmaniasis (CL), Mucocutaneous Leishmaniasis (MCL) and Diffuse Cutaneous Leishmaniasis (DCL) which typically range from self-healing but potentially disfiguring skin lesions (i.e. CL) or disseminated skin nodules (i.e. DCL), to severe damage to the nose and mouth mucosa (i.e. MCL). Visceral Leishmaniasis (VL) is a systemic disease and often lethal if untreated (Burza et al., 2018). Currently, these infections are managed clinically by chemotherapy, however drug toxicity and emerging resistance to front line treatments highlight a need for novel treatment options (Ponte-Sucre et al., 2017; Capela et al., 2019).

Hallmarks of genome instability (i.e. aneuploidy, CNVs and SNPs) are widespread in *Leishmania*, reported in natural isolates and laboratory populations (Reis-Cunha et al., 2018; Dumetz et al., 2018; Patino et al., 2019; Cupolillo et al., 2020). Like fungi and cancer cells (Sheltzer et al., 2011; Pfau and Amon, 2012; Lukow et al., 2021; Sah et al., 2021), some of these genomic rearrangements coincide with drug resistance and environmental adaptations (Dumetz et al., 2017; Dumetz et al., 2018; Patino et al., 2019), yet how *Leishmania* balance potentially beneficial instability whilst retaining genome fidelity is unknown. Furthermore, whether (or how) this plasticity directly contributes to the spectrum and severity of disease is unclear. Collectively, *in vitro* evidence points to DNA repair (Laffitte et al., 2014; Laffitte et al., 2016a), DNA repeats (Ubeda et al., 2008; Ubeda et al., 2014), unusual DNA replication dynamics and enhanced DNA replication stress as plasticity drivers (Damasceno et al., 2018; Damasceno et al., 2020a; Damasceno et al., 2020b) implying this phenomenon is multifactorial and intimately linked with specific features of the *Leishmania* genome and wider biological processes.

## 2 Repeated DNA sequences can catalyse *Leishmania* genome plasticity

In eukaryotes, repeated sequences of DNA can drive gene expression changes and genome diversification (reviewed by Biscotti et al., 2015; Kratochwil and Meyer, 2019; Brown and Freudenreich, 2021). In *Leishmania*, ~10% of the genome is populated with repetitive DNA, which is considerably less than predicated for the related pathogens *Trypanosoma brucei* (~20%) and *Trypanosoma cruzi* (~50%) (Pita et al., 2019). However, recent analyses implicate a wide variety of *Leishmania* DNA repeats catalyse their extreme genome plasticity (Ubeda et al., 2014; Bussotti et al., 2021).

Approximately 2000 low complexity Direct Repeats (DRs) and Inverted Repeats (IRs), named in relation to their genomic orientations, are present in the *Leishmania* genome. From these DNA repeats, ~3000-4000 unique and selectable extrachromosomal circular or linear amplicons are estimated to arise (Ubeda et al., 2014), originating from the genome and carrying potential fitness enhancing traits. Amplification is proposed to occur stochastically with subsequent changes to the abundance of beneficial amplicons leading to alterations in RNA levels under stressful environments, for instance following drug exposure (Ubeda et al., 2008; Leprohon et al., 2009; Ubeda et al., 2014; Laffitte et al., 2014; Bussotti et al., 2021). Broadly, the locations of DRs and IRs are syntenic across different *Leishmania* species (Dias et al., 2007; Ubeda et al., 2008; Ubeda et al., 2014) most (~68%) belonging to a family of extinct transposable elements (TEs), known as Short Interspersed DEgenerate Retroposons (SIDERs), that became expanded in *Leishmania*. Two subfamilies of SIDER elements have been described in these parasites: SIDER1 and SIDER2. Experimentally, SIDER elements can destabilise messengerRNA (mRNA) and may perform broader functions relating to the regulation of three prime untranslated regions (3’UTRs) (Bringaud et al., 2007; Smith et al., 2009; Müller et al., 2010; Requena et al., 2017), although further study is required to understand these roles. Nonetheless, no evidence suggests DRs or IRs perform functions outside of their described roles in extrachromosomal genome amplification.

**Figure 1:**
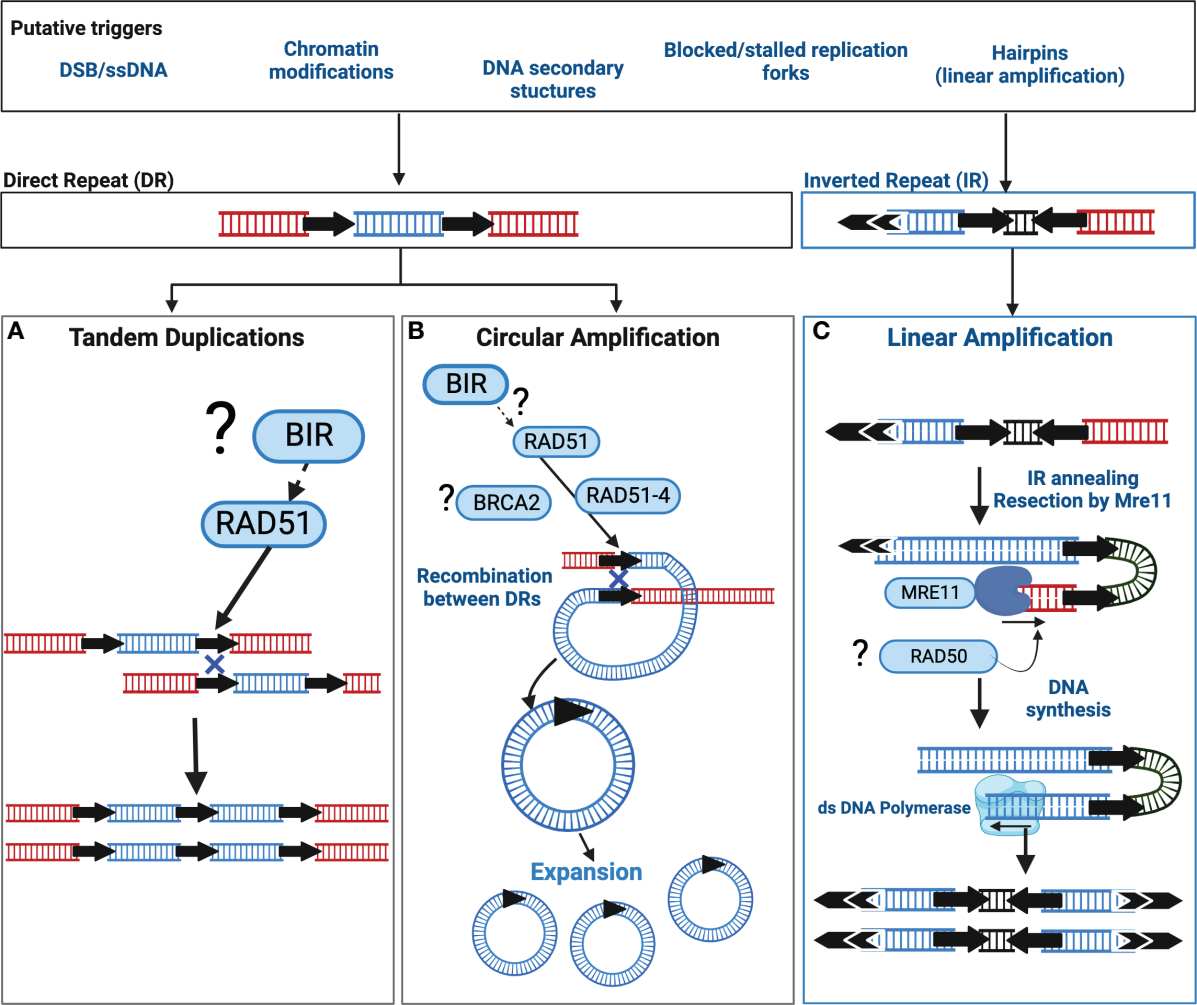
Putative models of extrachromosomal amplification in *Leishmania* driven by DNA Repeats. In *Leishmania*, extrachromosomal DNA amplification can be catalysed by either Direct Repeats (DRs) or Inverted Repeats (IR). Though the precise trigger(s) are unknown, putative sources of DNA instability are listed in the corresponding box above that may contribute to DNA amplification in *Leishmania*. Recombination reactions associated with DRs can result in tandem duplications and circular amplicons. The recombinase RAD51 facilitates a homology driven recombination between DRs that may result in **(A)** a tandem duplication, or **(B)** extrachromosomal circular amplicons. The mechanism driving tandem duplication events is unclear and may be the result of Break Induced Replication (BIR) or form an uneven exchange of genetic information between sister chromatids. Black arrows = DRs, (?) = the involvement of this factor or pathway requires experimental confirmation, Blue Cross indicates homologous recombination. **(C)** Linear amplification is driven through an annealing reaction between IRs. Here, the exonuclease activity of Mre11 may process a DNA lesion, for example a single strand break, or a hairpin structure formed due to DNA replication, after which the IRs anneal, and the DNA is replicated. Double arrows = telomeric sequences. Black arrows = IRs. Diagram adapted from (Laffitte et al., 2016b).

Current data supports two distinct pathways orchestrate *Leishmania* extrachromosomal amplification: one for linear amplification and one for circular amplification (summarised in **Figure 1**) however to date, neither pathway has been completely described. Extrachromosomal DNA circles, and tandem duplications in *Leishmania* exploit the activity of the recombinase RAD51 that facilitates a recombination reaction between DRs, subsequently leading to the formation of a circular amplicon or a duplication event (**Figure 1**). RAD51 is a key orchestrator of the homologous recombination (HR) pathway (Wright et al., 2018; Elbakry and Löbrich, 2021), required for double strand break (DSB) repair, thus the involvement of RAD51 is suggestive of unstable DNA or DNA injuries as catalysts. Additionally, RAD51 paralogues are also known regulators of RAD51 activity (Sullivan and Bernstein, 2018) and in *Leishmania*, RAD51-4, one of three *Leishmania* RAD51 paralogues, acts during circular amplification (Genois et al., 2015). Whether this role relates to the regulation of RAD51 activity remains untested. Direct interactions between *Leishmania* RAD51 and the mediator protein BRCA2 have also been experimentally confirmed but outside of the context of circular amplification (Genois et al., 2012). Thus far, we still lack key insights into three important events: 1) what triggers circular amplification, 2) what factors initiate amplification and, 3) what processes regulate amplicon abundance and consequently, their expression. Recent studies now shed light on some of these events (discussed below); nonetheless, wider identification and examination of circular amplification pathway members are still required.

The events initiating linear amplification are also largely elusive, though DSBs, single strand breaks (SSBs) and DNA hairpin structures are proposed triggers (see **Figure 1** for more details). In contrast, linear amplification does not rely on RAD51 or RAD51-4. Instead, the DNA repair enzyme MRE11 (Meiotic REcombination 11), a component of the Mre11-Rad50-Nbs1 (MRN) complex, plays a key role in facilitating annealing reactions between IRs (**Figure 1**); disrupting Mre11 activity impairs linear but not circular amplification (Laffitte et al., 2014).

Common to both is the co-option of DNA repair enzymes (and potentially wider pathways) supporting intrinsic DNA instability as a putative trigger. In agreement, the activities of RAD51 or Mre11 are not solely restricted to extrachromosomal amplification: Mre11 inactivation alone or in combination with RAD50 disruption is associated with chromosome translocations and broader instability (Laffitte et al., 2016a) whereas the loss of RAD51, *via* rapamycin induced LoxP excision, disrupts core chromosome duplication (Damasceno et al., 2020b). Indeed, the study by Damasceno and colleagues highlights DNA replication as potential contributor to this instability, with replication stress, a phenomenon that describes abnormal replication machinery progression, experimentally enhancing *Leishmania* genome diversity (Louradour et al., 2020) and driving subtelomeric duplication (Damasceno et al., 2020a). Whether the DNA repeats themselves are the source of instability (i.e prone to DNA breaks or secondary structures) requires testing. One other feature of these DNA repeats yet to be investigated is the relevance of their genomic positioning; DRs are dispersed more evenly across the chromosome, whereas IRs are concentrated at chromosome ends (subtelomere and telomere proximal regions) (Ubeda et al., 2014). Whether these sites impact upon the type of DNA amplicon is not known.

If, and how, linear amplicons are transmitted is undetermined, however circular DNA amplicons experimentally transmit *via* two distinct routes: 1) trans-generationally during cell division, 2) as part of the contents of extracellular vesicles (EV’s). During cell division, *Leishmania* daughter cells can inherit circular amplicons, but the processes that govern circular amplicon inheritance are undefined. In cancer cells, extrachromosomal circular DNA transmission appears to be random during cellular division (Lange et al., 2022), therefore it is possible the inheritance of circular amplicons in *Leishmania* is also random (Lange et al., 2022). Moreover, circular amplicons are typically lost once stressors are removed (Beverley et al., 1984; Ubeda et al., 2008; Leprohon et al., 2009), thus likely they pose a fitness cost in less restrictive circumstances. A second route of transmission emerged more recently, in which circular amplicons containing drug resistance genes were found within EVs, correlating with the emergence of drug resistant parasites in response when exposed to a specific compound (Douanne et al., 2022).

To date, all these experiments were performed using promastigotes, and currently it is unknown if these drug resistance genes re-integrate into the genome and/or are maintained after amastigote differentiation. Nevertheless, such findings could have significant impacts on our understanding of *Leishmania*- host and -vector interactions. Whether *Leishmania* utilise these amplification products to directly modulate their immediate extracellular environment and potentially alter disease progression, requires testing. Thus far, exposing immune cells to *Leishmania* EVs correlates with a Th2 directed anti-inflammatory response (da Silva Lira Filho et al., 2022) suggesting *Leishmania* excreted products can influence the host immune response. Indeed, in some human cancers, extracellular extrachromosomal circular DNAs have been reported in connection with altered disease outcome, acting as putative biomarkers of tumour severity (as reviewed by Li et al., 2022; Noer et al., 2022).

Additionally, the transmission of DNA amplicons could have consequences for species evolution. It is exciting to consider that mixed species infections of *Leishmania* provide opportunities for inter-species DNA transmission, and indeed such hybrids have been detected (Romano et al., 2014; Louradour et al., 2020). Currently, it is unknown whether circular or linear amplicons contribute. One final striking gap in our understanding, as alluded to previously, is how circular (and linear) DNA amplicons are copied. Whether similar processes duplicate the chromosomes and extrachromosomal DNA is unclear, or at which cell cycle stage these processes occur. The ability of *Leishmania* to duplicate exogenous sources of DNAs (i.e plasmids or cosmids of bacterial origins), suggests the replication pathway for extrachromosomal DNA is unlikely to rely on *Leishmania* specific sequences or factors (Papadopoulou et al., 1994).

Low complexity repeats, LDPR1, TATE and LINE elements are also found in the genome of *Leishmania* (Pita et al., 2019; Bussotti et al., 2021), yet their functions are understudied. Furthermore, 8 additional repetitive elements were linked to CNVs (Bussotti et al., 2021), mapping proximal to known CNV sites (~1 kb outside the variable region to ~ 150 bp within). Future studies will be key in deciphering their contributions to *Leishmania* genome variability.

## 3 Mosaic aneuploidy in the *Leishmania* genome

### 3.1 The origins of *Leishmania* aneuploidy

**Figure 2:**
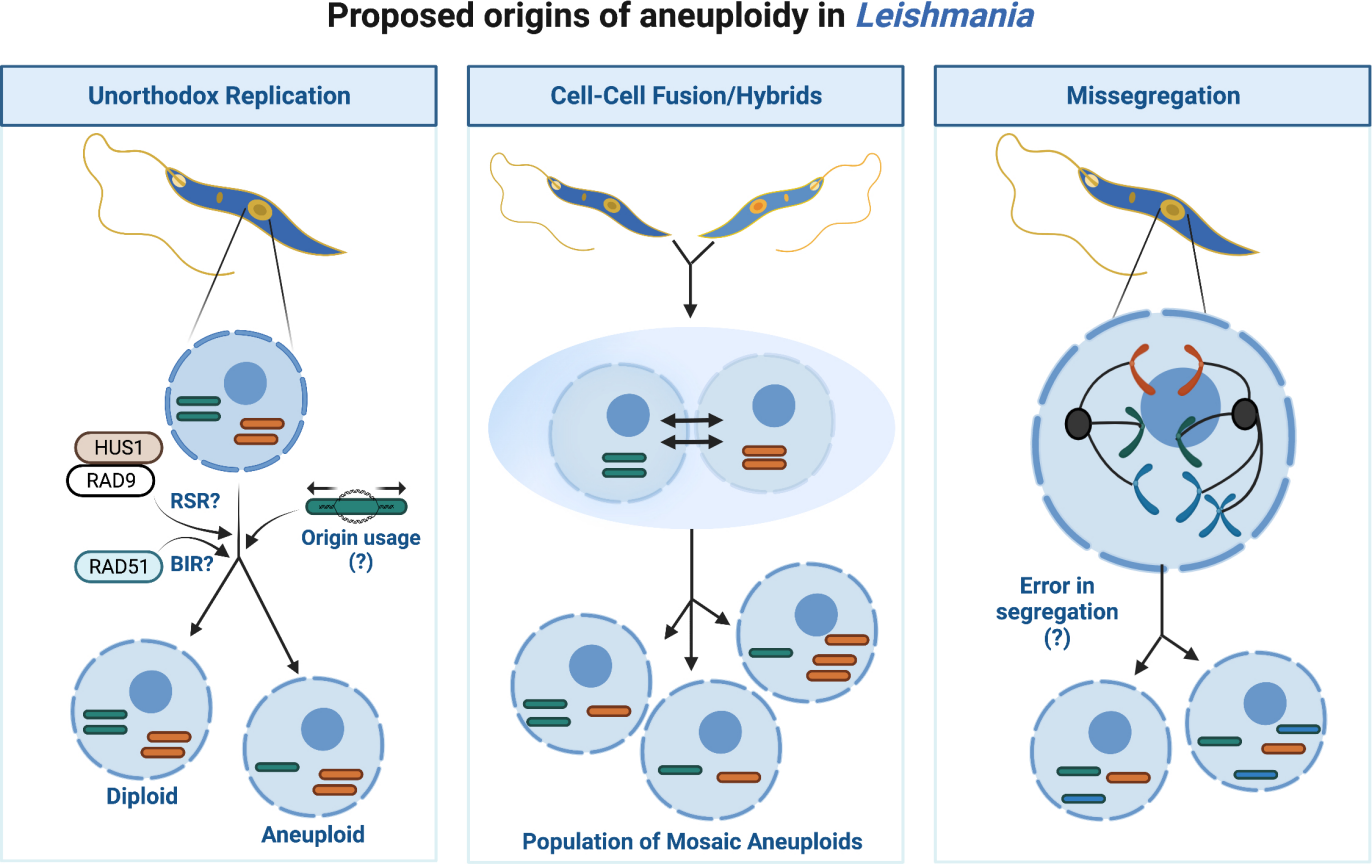
Proposed origins of aneuploidy in *Leishmania.* Aneuploidy in *Leishmania* may arise through several different processes or from a combination of events as illustrated. Unorthodox DNA replication may enhance opportunities for replication associated stress, for instance the potential usage of replication stress response (RSR) machinery for subtelomeric duplication and/or BIR to duplicate the chromosome cores may lead to under- or -over replication of chromosomes and thus aneuploid daughters. Cell-Cell fusions/hybrids may arise within the population leading to a fusion of cells each containing different chromosome numbers or putative hybrid events between different *Leishmania* species. Missegregation of chromosomes from mother cell to daughters may perpetuate aneuploidy, though the underlying biological processes permitting abnormal segregation are unclear.

Aneuploidy and CIN describe imbalances in chromosome numbers. Typically, CIN describes an inability to retain the same number of chromosomes from one division to the next, whereas aneuploidy explains a state of abnormal chromosome number. Though an aneuploid cell does not always experience CIN, often both coexist, particularly in cancers (Potapova et al., 2013). In humans, aneuploidy commonly correlates with early miscarriage (Ben-David and Amon, 2020), and developmental syndromes including Down Syndrome (Trisomy 21) (Antonarakis et al., 2020). Yet, in unicellular eukaryotes like yeast (Hose et al., 2020) and *Leishmania*, aneuploidy and CIN may enhance genome diversity. Disomy (i.e. two chromosome copies) likely predominates in *Leishmania*, however mosaic aneuploidy (variable aneuploidy states) is common *in vitro* and within natural populations, suggesting it is a constitutive feature of their genome (Rogers et al., 2011; Sterkers et al., 2011; Negreira et al., 2022). Why aneuploidy is frequent in *Leishmania* is unclear but like extrachromosomal amplification, varying chromosome number may provide an additional method of mRNA regulation by increasing DNA copies. In fact, correlations exist between chromosomal copies and gene expression for all chromosomes, except for chromosome 31 (Dumetz et al., 2017; Prieto Barja et al., 2017). Conversely, CNVs arising from aneuploidy do not always mirror protein abundance (Cuypers et al., 2022) suggesting additional layers of regulation operate, perhaps to mitigate wider effects due to haploinsufficiency or toxic overexpression of co-amplified genes. Besides providing populational variability, *Leishmania* could also use chromosomal duplication and loss to exclude whole chromosome variants leading to loss of heterozygosity (LOH), a process termed haplotype selection (Prieto Barja et al., 2017). During this process *Leishmania* cells may duplicate a disomic chromosome (chromosomes AB), becoming trisomic (chromosomes AAB), and lose the unwanted copy (chromosomes AA), reducing its heterozygosity. However, the relevance of this process to *Leishmania* evolution is poorly understood. Thus, the origins of aneuploidy in *Leishmania* are likely multifactorial, arising from lax chromosome segregation (i.e CIN), hybridisation *via* cell-cell fusions and from the unusual replication dynamics of the parasite, or a combination of these events (as summarised in **Figure 2**).

DNA replication in *Leishmania* (reviewed by da Silva et al., 2017; Damasceno et al., 2021) could contribute to aneuploidy in several ways. A unanimous model for *Leishmania* DNA replication has yet to be reached (Lombraña et al., 2016; Stanojcic et al., 2016; Damasceno et al., 2020a; da Silva et al., 2020), though current data supports replication preferentially initiates from a single site (an ‘origin’) per chromosome during early S-phase. Generally, the origin site is positioned at a transcription unit boundary (or strand-switch region: SSR), however why replication initiates here is still unknown. No defined sequence motifs have been identified at such sites; instead, the co-localisation of transcription associated epigenetic marks Base J (a modified thymidine) and acetylated Histone H3 (AchH3), in addition to the presence of the kinetochore protein KKT1 designate replication initiation permissive SSRs (Damasceno et al., 2020a). This unusual replication program may pose problems for chromosome duplication. For smaller chromosomes, firing a singular origin could accommodate complete duplication however, larger chromosomes may fail to duplicate prior to S-phase completion. Alternative ‘dormant’ origins could exist, as detected in *T. cruzi* (Calderano et al., 2015), however inducible deletion of RAD51 revealed a potential ‘origin-independent’ process may operate. Break Induced Replication (BIR), a mutagenic HR-like pathway which tackles single ended DSBs (reviewed by Kramara et al., 2018), has been proposed to complete *Leishmania* core chromosome duplication (Damasceno et al., 2020b). For subtelomeric sites, separate replicative processes appear to act, relying on replication stress response (RSR) machinery post-S phase (Damasceno et al., 2020a). Thus, the temporal organisation of *Leishmania* DNA replication may enhance opportunities for chromosomes to become over- or under-replicated. In support, cells in varying ‘somy’ states exist during mitosis, coinciding with the emergence of aneuploid daughters (Sterkers et al., 2011; Sterkers et al., 2014). Furthermore, DNA duplication involving DNA repair pathways (i.e. BIR) and post-S-phase synthesis correlates with enhanced mutagenesis in other eukaryotes (Ivanova et al., 2020 and reviewed by Saxena and Zou, 2022). Evidence of BIR or a BIR-like pathway during *Leishmania* DNA synthesis requires further testing however, together, these unusual replication dynamics could support frequent chromosome losses or gains and increased mutagenesis, particularly at subtelomeric sites, which are common instability ‘hotspots’. Repeated DNA and expanded gene families typically populate eukaryotic subtelomeres and, consequently, can undergo rapid evolution due these elevated levels of mutagenesis and recombination (Freitas-Junior et al., 2000; Linardopoulou et al., 2005; Rudd et al., 2007; Chen et al., 2018). The subtelomeres of *T. brucei* and *T. cruzi* harbour variable gene families that play key roles during host immune evasion (Ramirez, 2020; Sima et al., 2022), and thus are vital to parasite survival. Perplexingly, *Leishmania* subtelomeres, unlike *T. brucei* and *T. cruzi*, are devoid of variable gene families, thus why diversification would be required is unclear.

Aneuploidy could arise from cell-cell fusions (i.e hybridisation) in *Leishmania*. Cellular fusion with temporary tetraploidy (4 chromosome copies), followed by genome erosion with chromosomal loss was recently shown to occur in hybrids from the *Leishmania* close-related parasite, *T. cruzi* (Matos et al., 2022). Heterozygosity is rarer in natural isolates, relative to experimental strains; nonetheless, inter-, and intra-species hybrids exist (Volf et al., 2007; Romano et al., 2014; Louradour et al., 2020). It is possible interspecies hybridisation events, in addition or as an alternative to, extrachromosomal DNA transmission could explain the origin of certain *Leishmania* species. For instance, two chromosome fusion events in *Leishmania mexicana* may indicate genetic streamlining from the original parents. Moreover, a meiotic-like cycle may exist in *Leishmania* (Lanotte and Rioux, 1990; Weedall and Hall, 2015; Inbar et al., 2017; Inbar et al., 2019), and the generation of viable experimental hybrids can be enhanced following parasite exposure to genotoxins, indicating DNA repair plays a role in this process (Louradour et al., 2020; Ferreira and Sacks, 2022). However, exposure to genotoxic agents results in polyploid hybrids, which are different to the typical disomic hybrids observed in natural non-genotoxic exposed sandfly infections (Inbar et al., 2019). On the other hand, a mix of diploid, triploid and tetraploid hybrids were observed following *L. tropica* hybridisation *in vitro*, suggesting that polyploidy could arise even in the absence of genotoxic agents (Louradour et al., 2020; Ferreira and Sacks, 2022). Moreover, a recent study by Ferreira et al. (Ferreira et al., 2022), demonstrated the ability of *Leishmania* to generate self-hybrids in the insect vector. Indeed, the use of self-hybridisation could potentially limit the accumulation of deleterious mutations that could arise from rounds of asexual reproduction (Muller, 1964). That said, as certain meiotic cycle regulators appear absent from the genome, and no haploid intermediate stages have been identified in *Leishmania*, this cycle could be atypical as proposed previously (i.e. parasexual) (Sterkers et al., 2014). Irrespective, a meiotic-like process could explain chromosome shuffling and limited recombination events between parental chromosomes leading to altered chromosome dynamics in the offspring. Such a process may have important implications for mixed species infections, particularly if they occur in the mammalian host. It is still unclear whether genetic exchange occurs at this stage given the rarity of aneuploidy events in amastigote stages (Domagalska et al., 2019).

Lastly, imperfect chromosome segregation may generate CNVs in *Leishmania.* Less is known about the cell cycle checkpoints of *Leishmania* and the apparent absence of some Spindle Assembly Checkpoint (SAC) factors in the genome (Wheeler et al., 2019; Kops et al., 2020) could suggest lax or absent spindle checkpoint controls thereby supporting lenient spindle attachments, asymmetrical allotments, and potentially partial chromosome deletions. An alternative checkpoint exists during metaphase in procyclic (insect) forms of *T. brucei* which becomes instead activated in response to damaged DNA (Zhou et al., 2019) though in *Leishmania*, such a checkpoint remains undescribed.

Together, *Leishmania* aneuploidy and CNVs could arise from several sources, perhaps enhanced by this parasite’s unusual biology.

### 3.2 *Leishmania* aneuploidy is stochastic

Studying CNV regulation and its biological relevance in *Leishmania* is challenging. Foremost, we lack functional data for ~ 50% of the coding content of the genome, with less known about non-coding elements. Such gaps impair our ability to evaluate the consequences of aneuploidy events without subsequent targeted phenotyping. Secondly, the extreme malleability of the *Leishmania* genome often hinders basic reverse genetics approaches for phenotyping. Thirdly, the polycistronic transcription of functionally unrelated genes complicates how parasites balance beneficial dose alterations whilst mitigating toxic effects. Lastly, CNVs are often studied in the context of a phenotype, thus we likely lose resolution on the events initially promoting amplification or deletion prior to phenotype emergence. For instance, the detection and expansion of drug resistant phenotypes already threatens the clinical management of the disease. However, recent works by Negreira et al. (Negreira et al., 2022) and Bussotti et al. (Bussotti et al., 2021) are now refining our view on these processes by uncovering patterns of CNV that lead to parasite population heterogeneity associated with changes in gene expression and parasite evolution.

SCS used to study aneuploidy in *Leishmania* promastigotes by Negreira et al. produced several key findings. By comparing two independent clonal lines, one predominantly euploid (BPK081, clone 8) and one with variable somies (BPK282, clone 4), in *in vitro* cultured *L. donovani* promastigotes, a diversity of complex karyotypes was found co-existing within the population at any given time indicative of a genome under stochastic flux. Such diversity surfacing from both predominantly euploid and aneuploid founder populations reinforces this aneuploidy as stochastic. Their data supports an initial expansion of karyotype complexity that refines over time, leading to the emergence of more dominant (‘common’) karyotypes. This suggests that *Leishmania* steadily accumulate chromosome expansions in culture, which is a permissive environment. Subsequent alterations may further shape beneficial genomic changes. A small proportion of cells carrying rarer karyotypes persist in the population, perhaps because of the rich culture medium environment. Nonetheless, rare karyotypes could act as additional diversity reservoirs for overcoming subsequent bottlenecks including differentiation across lifecycle stages and vector or host entry.

One puzzling aspect of aneuploidy in *Leishmania* is the seeming preference for certain chromosomes to readily increase or decrease in copy, whilst others remain disomic or monosomic, at least in these two evaluated clones and their derived populations. Thus, restrictions presumably operate to limit supernumerary chromosomes. However, it could be possible to explain this effect as experimental limitations. Their data supports a model in which all chromosomes may possess the potential for amplification and additional selective pressures likely define which subset are frequently polysomic. Therefore, despite chromosome CNV itself being constrained, some underlying flexibility is retained if required (Negreira et al., 2022). It will be interesting to evaluate if the chromosomes consistently observed as disomic or polysomic by Negreira et al. will also maintain this pattern when other *Leishmania* populations or species are evaluated. Given ‘somy’ alterations often reverse if disadvantageous, aneuploidy likely imposes fitness costs for the parasite despite its frequency and seeming significance to *Leishmania* gene regulation. One surprising finding was the discovery of some parasites *in vitro* lacking entire chromosomes (i.e. nullisomic). Chromosome loss correlates with reduced genetic diversity within populations, and therefore counterintuitive for population diversification. Nullisomy is common in several plant species (i.e. wheat), often coinciding with the amplification of other homologous chromosomes to mitigate consequences of entire chromosome content depletion (Zhang et al., 2017). Whether true nullisomy naturally occurs in *Leishmania* is unclear but if supported, this strategy could serve as a ‘last-resort’ to remove survival-limiting genes under highly restricted environments. On the other hand, these nullisomic cells may arise from unbalanced cell division, and may lack long term viability.

Taken together, stochastic aneuploidy in *Leishmania* could represent a unique opportunity for genomic pre-adaption in the absence of stochastic alterations to transcription levels. These events may occur more freely in permissive conditions such as during *in vitro* culture and potentially within the sandfly environment.

## 4 Do epistatic pathways direct chromosome and gene copy number?

A routinely cited example of chromosome polyploidy in *Leishmania* is chromosome 31 of *L. major* and all other evaluated species to date (Rogers et al., 2011). Why chromosome 31 is apparently always supernumeric in copy number is unknown. However, recent data suggests this polyploidy may correlate with increased chromosome 15 amplification (Negreira et al., 2022), suggestive of unknown physical and/or functional inter- and intra-chromosome interactions. Indeed, in *T. brucei* chromosome interactions regulate transcription and splicing of the variant surface glycoprotein (VSG) required for host immune evasion (Faria et al., 2021) highlighting the importance of these events in host evasion. Yet, we currently lack evidence linking chromosome interactions to gene expression changes in *Leishmania* as detailed maps of such interactions are still to surface.

One emerging explanation to describe these correlative ploidy changes between chromosomes pertains to the non-coding RNAs (ncRNAs) content (Bussotti et al., 2021; Negreira et al., 2022). Although ncRNAs do not encode proteins, they are key regulators of cellular metabolism (Cech and Steitz, 2014). In *Leishmania*, the ‘RNAome’ may contain upwards of 12,000 ncRNAs per species but limited studies have functionally characterised their activities (Ruy et al., 2019; Fort et al., 2022). Now, links between ncRNAs and parasite development suggest these elements do directly regulate key parasite processes, for example the recent description of a long ncRNA required for differentiation to the quiescent, transmissible form (the ‘stumpy’ form) of *T. brucei* (Guegan et al., 2022) or the variable expression of ncRNAs across the *Leishmania* lifecycle (Ruy et al., 2019). Whether ncRNAs play roles in *Leishmania* genome plasticity is unknown, though thus far, small nucleolar RNA (snoRNAs), transfer RNA (tRNAs) and ribosomal RNA (rRNAs) appear to associate with chromosome polyploidy and gene CNVs (Bussotti et al., 2021; Negreira et al., 2022), though the natures of these relationships require further clarification. Nevertheless, differential snoRNA expression in *Leishmania* correlates with rRNA changes and the production of modified ribosomes, in turn altering mRNA turnover and translation (Piel et al., 2022). Together, these data could explain the lack of a defined relationship between the coding content of co-amplified chromosomes.

One study exploring these effects in culture adapted *L. infantum* promastigotes uncovered evidence of putative relationships between co-amplified genes and those of similar functionalities (Bussotti et al., 2021), attributing their findings to an underlying and functional ‘epistatic’ network. Epistasis is a phenomenon that broadly describes the outcome of a mutation or mutations as functions of the genetic background they appear in. For example, a mutation of a gene which enhances gene expression in one genetic background, may instead have differing effects in another (Domingo et al., 2019). In *Leishmania*, the spontaneous deletion of an 11kb region containing an essential NIMA-related kinase led to viable *in vitro* promastigotes suggesting an unknown compensatory method(s) operates, independently, to limit potentially fatal genomic alterations. Therein, the authors reported an increased abundance of 350 transcripts including ncRNA elements and metabolic enzymes in their deletion mutants (Bussotti et al., 2021). Similarly, it was demonstrated in another recent study that non-targeted deletions can be induced as compensatory mechanisms in *Leishmania* when targeting an essential gene (Alpizar-Sosa et al., 2022). Thus far, definitive evidence of epistatic interactions in *Leishmania* is still required. These data are frequently challenging to interpret and the wider implications of such interaction networks in the context of an infection must be investigated.

In summary, during early adaptions, flexible gene dosage variation, that may include non-coding elements, could alter translation and RNA stability regulation thereby regulating expression rapidly. Later (and likely more stable) adaptions appear to require more extensive alterations to genomic content.

## 5 Concluding remarks and future directions

**Figure 3:**
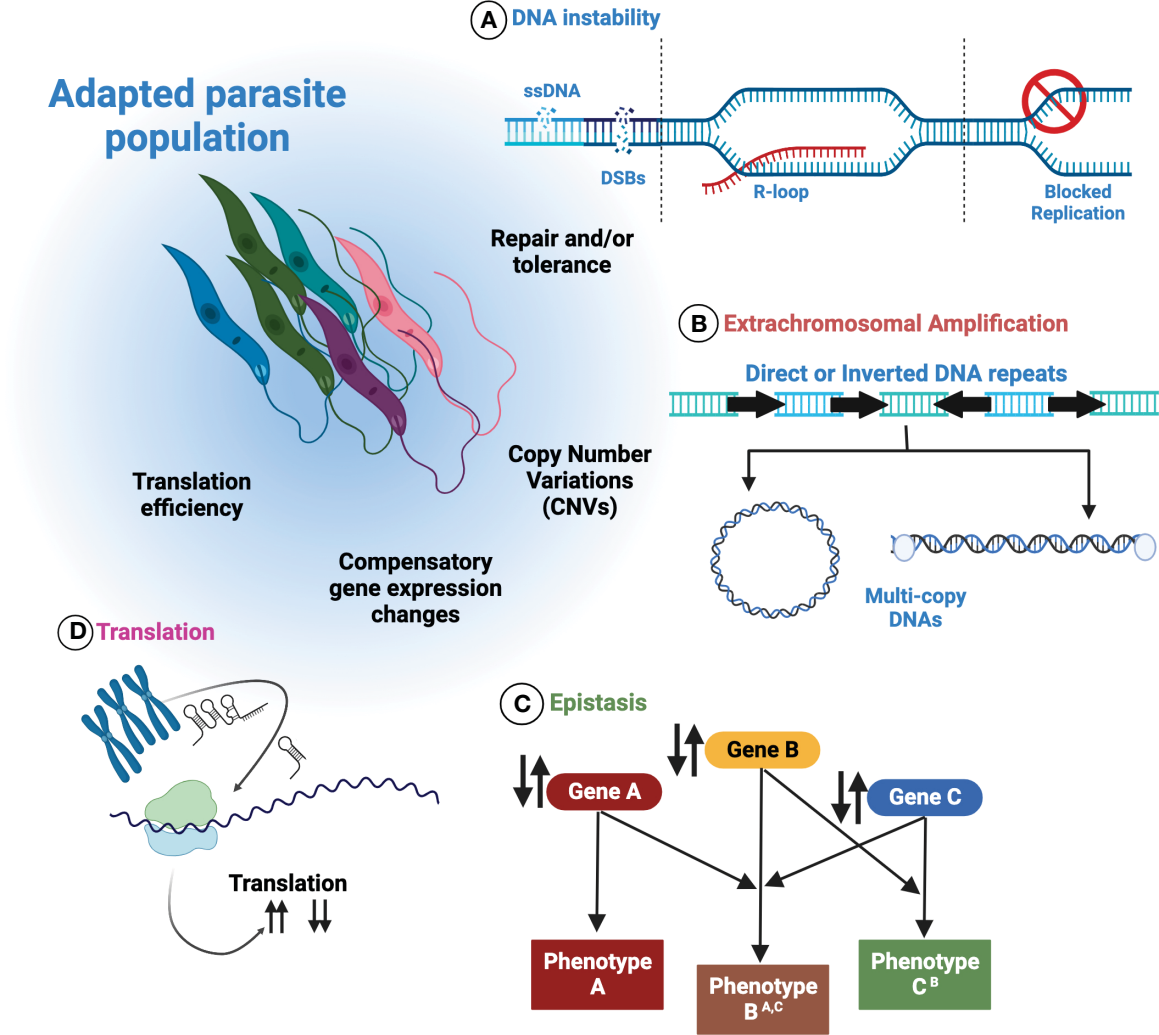
Control over genome plasticity in *Leishmania* is multifactorial. Numerous processes are thought to contribute to *Leishmania* genome plasticity. **(A)** Inherent DNA instability may be a key source of genome plasticity events in *Leishmania*. How these events are repaired and/or tolerated may offer deeper insights into how they are exploited by *Leishmania*. **(B)** By increasing or decreasing the abundance of extrachromosomal amplicons, *Leishmania* can modulate the corresponding mRNA levels in response to changing environments. **(C)** Epistatic interactions may operate in *Leishmania*, allowing compensatory mutations or genomic rearrangements to arise. **(D)** The putative regulation of translation by non-coding RNAs may permit the modulation of translation efficiency.

Possession of a plastic genome presents *Leishmania* with benefits and challenges. Likely arising from multiple sources (summarised in **Figure 3**), the ability of *Leishmania* to maximise and harness stochastic instability, generated by core biological processes, may favour the frequent discovery of beneficial traits in harsh and changing environments. Adjustments to the abundance of favourable genes, followed by putative regulatory interactions by ncRNAs, DNA modifications and chromatin alterations could allow *Leishmania* to fine-tune gene expression further by adapting translation efficiency. Moreover, the recent discovery of extrachromosomal DNAs within *Leishmania* EVs provides opportunity for the population-wide dissemination of fitness enhancing traits, offering naïve individuals a means of survival, and putatively maximising the persistence of the infection. Similarly, DNA exchanges in mixed species infections, for instance in the insect vector, may contribute to species diversification through the exchange and incorporation of amplified DNA from others.

Whilst the related pathogens *T. brucei* and *T. cruzi* can utilise variable antigen gene families to evade host defences, no such strategy appears to operate in *Leishmania*. Thus, a genetically diverse population of parasites with flexible, and rapidly evolving genomes may offer an alternative strategy for overcoming host defences. Whether such extensive diversity arises in the context of a clinical infection requires further experimentation given aneuploidy appears rarer in the mammalian stage parasites (amastigotes), and to some extent, in naturally isolated promastigotes. Technical limitations often thwart direct investigations in amastigotes from clinical isolates, namely poor sample size leading to an inevitable passage through mice or into *in vitro* culture. That said, it is possible that the exclusively intracellular lifecycle of amastigotes may impose fewer extreme demands for genetic plasticity.

In contrast, a genome under constant, stochastic flux is problematic. Too many random alterations could impede survival under restrictive environments, for instance within neutrophils or macrophages. Toxic rearrangements, persistent damage, and the irreversible loss of genetic information are serious consequences of unregulated genome instability and may compromise the parasite population in the face of further stressors. Thus, a deeper understanding of how *Leishmania* regulate their genome composition is crucial as currently *Leishmania* genome plasticity is a key barrier to the development of novel compounds for the treatment of leishmaniasis. Finally, even less known about the impact of these genomic changes on the host and subsequent future infections.

## Author contributions

All authors contributed equally to the writing and preparation of the manuscript and to the figure design.
